# The impact of extended half-life versus conventional factor product on hemophilia caregiver burden

**DOI:** 10.1007/s11136-018-1792-9

**Published:** 2018-02-01

**Authors:** Carolyn E. Schwartz, Victoria E. Powell, Jun Su, Jie Zhang, Adi Eldar-Lissai

**Affiliations:** 1 DeltaQuest Foundation, Inc., 31 Mitchell Road, Concord, MA 01742 USA; 2 Departments of Medicine and Orthopaedic Surgery, Tufts University Medical School, Boston, MA USA; 3 Bioverativ Therapeutics Inc., Waltham, MA USA; 4 Biogen, Cambridge, MA USA

**Keywords:** Hemophilia, Caregiver, Burden, Impact, Extended half-life factor product, Hemophilia Caregiver impact measure

## Abstract

**Introduction:**

Extended half-life factor products have reduced annualized bleeding rates in hemophilia patients. The impact of extended half-life versus conventional factor products on hemophilia caregiver burden has not been investigated. This study aimed to evaluate caregiver burden in extended half-life versus conventional factor products for hemophilia A and B.

**Methods:**

This cross-sectional web-based study of caregivers of people with hemophilia A or B was recruited from a panel research company and by word of mouth. Participants completed the Hemophilia Caregiver Impact measure, the PedsQL Family Impact Module (PedsQL), and the Work Productivity and Activity Impairment Questionnaire (WPAI). We also collected demographic, insurance coverage, and medical information related to the hemophilia patient(s). Burden differences were assessed using linear regression and matched cohort analyses.

**Results:**

The sample (*n* = 448) included 49 people who were caring for people on extended half-life factor products. Worse caregiver burden was associated with more infusions per week and more bleeds in the past 6 months. Regression analyses suggested that caring for someone who is on a extended half-life factor product is associated with lower emotional impact (*β* = − 0.11, *p* < 0.05, Adjusted *R*^2^ = 0.06), and shows a trend association with lower practical impact (*β* = − 0.09, *p* < 0.10, Adjusted *R*^2^ = 0.05). The matched cohort analysis also revealed that people on extended half-life factor product had lower Emotional Impact and Practical Impact scores (*t* = − 2.95 and − 2.94, respectively, *p* < 0.05 in both cases). No differences were detected on the PedsQL or the WPAI.

**Conclusion:**

The reduced required frequency of factor product infusions of extended half-life factor products appears to reduce the emotional distress and practical burden of caregiving. Future work should evaluate the longitudinal impact.

## Introduction

Hemophilia A and B are rare inherited bleeding disorders characterized by the deficiency of coagulation factors [1, 2]. Hemophilia A is a deficiency in factor VIII, whereas hemophilia B is a deficiency in factor IX [3]. Affecting an estimated 1 in 5000 (hemophilia A) and 1 in 30,000 (hemophilia B) male live births, the conditions can range from mild to severe [4, 5]. In both types of hemophilia, there is a known family history in about 70% of cases; about 30% of cases are thought to be the result of a spontaneous mutation [3]. In early infancy, hemophilia A or B is often identified by spontaneous hemorrhages and confirmed with a laboratory test [6]. Hemophilia A comprises 80–85% of hemophilia cases in the US, whereas hemophilia B comprises 15–20%. In patients with severe factor deficiencies (factor level less than 1%), bleeding of the ankle, knee, and elbow joints are extremely common [3]. Compared to hemophilia A patients, hemophilia B patients exhibit less severe bleeding and have better long-term outcomes, such as a lower likelihood of arthropathy [7]. As the person with hemophilia grows older, recurrent hemathroses and soft-tissue hematomas lead to severe arthropathy and joint contractures [8]. The resulting chronic pain and disability can have short- and long-term effects on physical, social, and emotional functioning [9, 10], as well as direct and indirect costs such as the impact of hemophilia caregiving on work productivity [11–14]. Further, patients may develop hypersensitivity reactions to factor replacement products (i.e., inhibitors), which occurs in about 25% of hemophilia A patients and 5% in hemophilia B patients [3], although inhibitors are less prevalent in hemophilia B, they can be more clinically significant, and can develop into nephrotic syndrome [3]. Fortunately, immune tolerance induction programs have permitted the eradication of inhibitors in two-thirds of patients [15, 16].

The development of recombinant technology led to the advent of safer factor concentrates that can not only be used to treat bleeding symptoms at home, but also for home-based infusion of factor product [3]. Two recent advances in hemophilia treatments have revolutionized hemophilia care [8]. First, the availability of recombinant factor products has enabled a preventive treatment approach, known as prophylaxis. Prophylactic treatment has notably improved the day-to-day management of hemophilia [17, 18]. It is particularly recommended for patients with severe forms of hemophilia because it improved patient outcomes [19]. Only about half of current patients use a prophylactic regimen, rather than infusing in reaction to a bleeding event (known as episodic or on-demand treatment).

The second major advance is the 2014 introduction of extended half-life factor products. These products have altered the landscape of hemophilia [8] by dramatically improving the management of acute bleeding by improving trough factor levels. They also provide a more convenient infusion schedule because fewer treatments per week are required [20]. Accordingly, they may facilitate better adherence. Extended half-life factor products are composed of a single molecule of rFVIII or rFIXFc, for hemophilia A or B, respectively, which is genetically fused to the IgG1 Fc domain, using Fc fusion technology. The Fc domain of these fusion proteins binds to the FcRn receptor in the endosome, delaying lysosomal degradation and thereby extending the half-life [21, 22]. The documented reductions in annual number of infusions and annualized bleeding rates in hemophilia patients [23, 24] hold great promise for reducing short- and long-term morbidity in patients. Preliminary results of extension studies from the pivotal open-label clinical trials evaluating extended half-life factor products suggest that patients evidence improvements in quality of life over time [25–28].

Despite the advantages of extended half-life factor products, there are barriers to changing from conventional factor product regimens. As patients are not required to switch to extended half-life products, there are barriers related to hesitancy to change something that works despite the inconvenience; patients and/or parents may be wary of the disruptive effect of switching products [20]. From the provider’s perspective, the extended half-life factor products have accentuated the variability of patient half-life, and have led to uncertainty about optimal dosing and monitoring strategies [20]. Further, there is the concern that switching from conventional to extended half-life factor products will risk inhibitor development (i.e., an autoimmune response to the factor product), although early results suggest there is no such risk [20].

Despite all of these advances, the need to infuse the hemophilia patient multiple times per week remains a major impediment to patient and caregiver quality of life. Parents are placed in a caregiving role as soon as the diagnosis is known. The need to infuse the hemophilia patient multiple times per week remains a major impediment to both patient and caregiver quality of life, particularly when the patient is a young child [29]. Caregiver quality of life is also impacted by the physical, social, and emotional strains of caregiving [9, 30–33]. Caregivers may confront distressing feelings of guilt or denial, and may not always be able to recognize bleeding symptoms. Hemophilia can also impact the family’s financial resources, not only due to the cost of care but also because of the demands that affect the caregivers’ ability to work and even to plan their day, week, or month.

Although initial estimates of outcomes of extended half-life factor products suggest that they are efficacious and safe [20, 23, 24, 34], more information is needed on their impact on quality of life. Since caregivers are greatly impacted by hemophilia, it would be worthwhile to know if such treatments also have a beneficial effect on caregivers of people with hemophilia. Research to date has not addressed the impact of extended half-life factor products on caregiver burden. This is a highly relevant hemophilia outcome, given the abovementioned salient impact of hemophilia on the caregiver(s). Accordingly, the purpose of the present study was to evaluate caregiver burden in extended half-life versus conventional factor products for Hemophilia A and B.

## Methods

### Design and sample

This cross-sectional study included caregivers of people with hemophilia A or B. Eligible study participants were fluent in English. Only one caregiver per family unit was allowed to participate in the study.

Recruitment. Participants were recruited from several sources. We began with participants from the hemophilia panel of Rare Patient Voice, LLC., and with caregivers from the item pretesting phase of the project [35]. We then utilized the *snowball technique* for enhancing participant accrual. This technique involves asking study participants to refer other eligible potential participants from their network of hemophilia-related friends and acquaintances. A natural outgrowth led to reaching out nationwide to chapters of hemophilia advocacy organizations, such as the National Hemophilia Foundation. Finally, we emailed Nurse Coordinators at Hemophilia Treatment Centers nationwide using a spreadsheet downloaded from the Centers for Disease Control website. This email provided information about the study objectives, eligibility criteria, and time commitment.

### Procedure

The study protocol was reviewed and approved by the New England Independent  Review Board (NEIRB #14-422). All procedures followed were in accordance with the ethical standards of the responsible committee on human experimentation (institutional and national) and with the Helsinki Declaration of 1975, as revised in 2008. This web-based study was administered using the HIPAA-compliant, secure SurveyGizmo engine (http://www.surveygizmo.com). We followed study procedures described by Dillman’s Tailored Design Method [36] to yield a maximal response rate. Dillman’s method spells out detailed descriptions of each step of sample recruitment to yield robust response rates. Maintaining a robust response rate is necessary for the external validity (i.e., generalizability) of the study findings. The Dillman method involves specific steps for personalizing study materials, providing motivating reasons for participation, paying attention to ease of use of survey interfaces, and optimal timing and content of follow-up reminders [36].

#### Incentive payments

All participants were paid $75 for participation in this cross-sectional survey. We offered a $5 incentive payment to those who referred eligible study participants who then completed the survey.

### Measures

The  Hemophilia Caregiver Impact (HCI) measure [30] is a validated 36-item caregiver-reported measure assessing the personal impact associated with caring for people with hemophilia. Responders were asked to complete the survey based on their experience in the past 6-months. The measure has eight domains: seven subscales assess relevant negative aspects of caregiver impact and one subscale assesses positive aspects of caregiving. The negative-impact subscales include (1) Practical Impact, which assesses the impact of ordering supplies, medical appointments, and travel to the hospital; (2) Symptom Impact, which assesses the impact of witnessing/suffering from the care recipient’s pain and caregiver worry and distress related to the hemophilia patient’s symptoms; (3) Social Impact, which assesses the impact of hemophilia on the family and spouse/partner relationships; (4) Physical Impact, which assesses the impact of hemophilia caregiving on the caregiver’s physical functioning/symptoms; (5) Emotional Impact, which assesses the impact of hemophilia caregiving on the caregiver’s emotional functioning/symptoms; (6) Lifestyle Impact, which assesses the impact of hemophilia caregiving on the caregiver’s/family’s discretionary activities, such as time for self, exercise; and (7) Financial Impact, which assesses the impact of hemophilia on the family’s financial status and work-related function. The positive-impact subscale comprises Positive Emotions, which assesses positive aspects of caregiving related to the sense of purpose and self-worth. Two summary scores can be used: a Burden Summary score and a Positive Emotions score. Higher scores on the negative aspects subscales indicate worse burden, whereas higher scores on the Positive Emotions score indicate more positive aspects of caregiving. For full details about the psychometric characteristics of the HCI, see [30]. In addition to the HCI, other person-reported measures included the PedsQL Family Impact Module [37] and the Work Productivity and Activity Impairment Questionnaire. The PedsQL Family Impact Module is a 36-item generic caregiver burden measure that contains subscales for the caregiver’s physical, emotional, social, and cognitive functioning; communication; worry; daily activities; and family relationships. The measure can be scored to yield a Parent HRQL Summary Score using the 20 items from the physical, emotional, social, and cognitive functioning subscales, and a Family Functioning Summary Score using the eight items from the daily activities and family relationship subscales [37]. Scores are first reverse-scored and linearly transformed to a 0–100 scale, and then summed [38]. High scores on the Peds QL indicate better functioning [38]. The nine-item hemophilia-specific Work Productivity and Activity Impairment Questionnaire was used to assess impact of hemophilia caregiving on work [39]. The measure was scored to reflect the percent of work time impaired due to health (i.e., sum of items querying work impairment due to health over total hours missed and actually worked, multiplied by 100) [39]. High scores on the Work Productivity and Activity Impairment Questionnaire reflect worse work impairment [39]. We also collected demographic, insurance coverage, and medical/treatment information related to all of the hemophilia patient(s) for whom the person was providing care.

### Statistical analysis

Correlation analyses examined the relationship between HCI scores and clinical variables, such as the frequency of infusions and bleeds. T-tests or Fisher’s Exact tests compared the caregivers whose care recipients used extended half-life versus conventional factor products. To evaluate caregiver burden in extended half-life versus conventional factor products, a radar plot displayed unadjusted mean scores by group. Based on the radar plot, we selected three HCI subscales and the Burden Summary score to compare in a subsequent series of hierarchical regression analyses to examine the impact of extended half-life factor products as compared to conventional factor products. Selecting a limited number of subscales also reduced the Type I error rate. We also examined group differences on the PedsQL subscales and WPAI. People on immune tolerance induction (ITI) protocols and caregivers caring for multiple hemophilia patients who were on both extended half-life and conventional factor products were excluded from analysis. For the 112 caregivers providing care to more than one person with hemophilia, we created a summary score for each variable describing their caregiving role or their care recipients. This score would generally be the highest value within their group of care recipients. Thus, if a caregiver provided caregiving support for at least one person who had inhibitors, the caregiver would be included in the “inhibitor” group. This approach captured the maximal burden represented by their care recipients.

We began by evaluating the distribution of the dependent variables and then computing models predicting the following dependent variables: the Practical Impact, Emotional Impact, and Positive Emotions HCI subscales or the Burden Summary score; the PedsQL Parent Health-Related Quality of Life (HRQL) Summary, Family Functioning, and Total Scores; and the WPAI Work Impairment Due to Health score. The following covariates were examined: caregiver age, gender, and education; number of years caring for person(s) with hemophilia; type of hemophilia, whether the person(s) has/had inhibitors; whether the person(s) is/are on prophylaxis treatment; and type of health insurance. We kept all covariates in subsequent models, and then examined the impact of using an extended half-life factor product by adding a dummy variable. Regression diagnostics were checked to ensure that none of the assumptions of regression modeling were violated. If heteroscedasticity was detected, we evaluated the impact of log-transforming the dependent variable and examined weighted least squares models. Finally, we examined the value of adding a covariate reflecting whether the caregiver was a parent of the patient to whom s/he provided caregiving support.

To improve the comparability between the two groups (i.e., extended half-life caregivers versus conventional caregivers) and to increase the likelihood of the results reflecting a true causal difference, we performed matched cohort analysis using the Stata “teffects” macro and nearest neighbor matching [40]. The matched cohort analysis allowed for a more sensitive comparison of the two groups by reducing ‘noise’ in the comparison (i.e., reducing heterogeneity of the comparison groups). Cohorts were matched based on: (1) whether the care recipients were on a prophylaxis regimen; (2) whether they had inhibitors; (3) the caregiver’s age; and (4) the number of years the caregiver has provided caregiving support. The former two were exact-matched and the latter two were nearest neighbor-matched and the models were bias-adjusted. By matching the two comparison groups on the four relevant covariates, these covariates no longer need to be included in the statistical model, thereby reducing degrees of freedom used and power lost. In the context of matched cohort analysis, “nearest neighbor” refers to accepting “matches” that are not exact but relatively close. For continuous variables (in our case: caregiver age and number of years providing caregiving support), slight differences would be expected and acceptable; but they are “bias-adjusted,” meaning that a linear function of the specified covariates is used to correct a bias that exists when matching continuous covariates [40].

Statistical analyses were implemented using Stata 14 [41]. Cohen’s [42] criteria for small, medium, and large effect sizes for correlational analysis and regression models were used to interpret study findings.

## Results

### Sample accrual

The web-based survey was implemented for 5 months. Informed consent was obtained from all patients for being included in the study. Following Dillman [36] steps for personalized outreach to participants and follow-through with the snowball technique, we found that participants’ referrals had a substantial impact on recruitment, reaching out to hemophilia chapters made some difference to increase recruitment, but reaching out to nurses at hemophilia treatment centers did not increase recruitment. We collected personal identifiers that ensure the participants completed the survey only once.

### Sample characteristics

Table 1 shows sample characteristics for the caregiver participants included in the analyses, and Table 2 shows the characteristics of their care recipients. The analytic sample included 448 caregivers, of whom 266 were recruited from Rare Patient Voice LLC and 182 were recruited by word of mouth. Approximately 11% of the sample provided caregiving support to people on extended half-life factor products.

**Table 1:** Caregiver sample characteristics

	Whole sample (*n* = 448)	Extended half-life factor product (*n* = 49)	Conventional factor product (*n* = 399)	Test statistic comparing extended half-life versus conventional factor product groups	*P* value
Caregiver age					
Mean (SD)	39.22 (8.70)	38.47 (8.55)	39.31 (8.72)	*T* = 0.5585	0.577
Caregiver gender					
Male (%)	11%	8%	12%	Fisher’s exact = 0.623	0.634
Female (%)	88%	92%	88%		
Missing (%)	0%	0%	0%		
Caregiver education					
High school or less (%)	14%	14%	14%	Fisher’s exact	0.210
Some college (%)	39%	43%	38%		
College (%)	31%	37%	30%		
Graduate degree (%)	16%	6%	17%		
Race					
American Indian or Alaska Native (%)	3%	4%	3%	Fisher’s exact	0.643
Middle Eastern (%)	1%	0%	1%	Fisher’s exact	1.000
South Asian (%)	1%	0%	1%	Fisher’s exact	1.000
Other Asian (%)	3%	0%	3%	Fisher’s exact	0.378
Black or African American (%)	8%	8%	8%	Fisher’s exact	1.000
Native Hawaiian or Pacific Islander (%)	1%	0%	1%	Fisher’s exact	1.000
Caucasian (%)	81%	88%	80%	Fisher’s exact	0.249
Marital status					
Never married (%)	8%	6%	8%	Fisher’s exact	0.717
Married (%)	73%	73%	73%		
Cohabitation/domestic partnership (%)	4%	6%	4%		
Separated (%)	3%	6%	3%		
Divorced (%)	10%	8%	11%		
Widowed (%)	1%	0%	1%		
Missing (%)	0%	0%	1%		
Number of children					
Mean (SD)	1.93 (1.20)	1.92 (1.34)	1.93 (1.18)	*T* = 0.0493	0.961
Number of children with hemophilia					
0 (%)	15%	18%	15%	*T* = 1.0836	0.279
1 (%)	67%	69%	67%		
2 (%)	15%	10%	15%		
3 (%)	2%	2%	2%		
4 (%)	1%	0%	1%		
Number of people caring for with hemophilia					
1 (%)	77%	84%	76%	*T* = 1.2104	0.227
2 (%)	19%	14%	20%		
3 (%)	3%	2%	3%		
4 (%)	1%	0%	1%		
5 (%)	0%	0%	0%		
Relationship to care recipient					
Son (%)	75%	78%	74%	Fisher’s exact	0.141
Daughter (%)	2%	0%	2%		
Children (%)	14%	6%	15%		
Brother (%)	0%	0%	0%		
Other family member (%)	6%	14%	6%		
Multiple family members (%)	3%	2%	3%		
Number of years caring for patient					
Mean (SD)	10.45 (6.95)	10.80 (6.42)	10.41 (7.01)	*T* = − 0.3669	0.714
Insurance type^a^					
Private (%)	73%	65%	74%	Fisher’s exact	0.232
Medicare, Medicaid, CHAMPUS, HIS, Supplemental (%)	26%	29%	26%	Fisher’s exact	0.732
Does not have insurance (%)	5%	10%	4%	Fisher’s exact	0.067

^a^Percentages may add up to more than 100 because people can have more than one type of insurance

**Table 2:** Care recipient sample characteristics

Characteristics of hemophilia care recipients	Whole sample (*n* = 572)	Extended half-life factor product (*n* = 60)	Conventional factor product (*n* = 512)	Test statistic comparing extended half-life versus conventional factor product groups	
Type of hemophilia					
A (%)	77.8%	61.7%	79.7%	Fisher’s exact	**0.003**
B (%)	22.2%	38.3%	20.3%		
Receiving prophylactic treatment					
Yes (%)	72.6%	83.3%	71.3%	Fisher’s exact	**0.026**
Past inhibitor					
Yes (%)	10.8%	3.3%	11.7%	Fisher’s exact	**0.046**
Time on current factor product (years)					
Mean (SD)	6.59 (5.55)	1.36 (1.80)	7.20 (5.51)	*t* = 7.9452	**0.000**
Number of infusions per week					
Mean (SD)	3.20 (1.98)	1.83 (1.02)	3.39 (2.01)	*t* = 5.3751	**0.000**
Age					
Mean (SD)	12.98 (12.15)	15.68 (14.61)	12.66 (11.80)	*t* = − 1.8267	0.068
Gender					
Male (%)	91.4%	90.0%	91.6%	Fisher’s exact	0.623
Female (%)	8.4%	10.0%	8.2%		
Missing (%)	0.2%	0.0%	0.2%		
Severity					
Mild (%)	11.5%	8.3%	11.9%	Fisher’s exact	0.290
Moderate (%)	15.7%	10.0%	16.4%		
Severe (%)	72.0%	81.7%	70.9%		
Missing (%)	0.7%	0.0%	0.8%		
Number of bleeds in the last 6 months					
Mean (SD)	3.95 (5.78)	2.98 (3.50)	4.07 (5.98)	*t* = 1.3529	0.177
Number of infusions per month for on demand treatment					
Mean (SD)	1.60 (3.40)	0.63 (1.41)	1.66 (3.47)	*t* = 0.8324	0.407
Clotting factor products (%)					
Advate	41.08			NA	
Adynovate	1.05			NA	
Alprolix	3.67			NA	
Benefix	14.86			NA	
Eloctate	5.24			NA	
Helixate FS	3.5			NA	
Hemofil	0.17			NA	
Ixinity	0.52			NA	
Kogenate FS	9.62			NA	
Monoclate	0.35			NA	
Mononine	0.52			NA	
Novoeight	2.8			NA	
Recombinate	2.45			NA	
Rixibis	1.57			NA	
Xyntha	2.97			NA	
Other	9.09			NA	
Clotting-promoter medications					
Amicar (%)	56.1%	58.3%	55.9%	Fisher’s exact	0.781
DDAVP (%)	2.8%	3.3%	2.7%	Fisher’s exact	0.678
Feiba (%)	3.9%	3.3%	3.9%	Fisher’s exact	1.000
Novo 7 (%)	5.6%	3.3%	5.9%	Fisher’s exact	0.563
Stimate (%)	6.8%	5.0%	7.0%	Fisher’s exact	0.787
Current inhibitor					
Yes (%)	8.2%	6.7%	8.4%	Fisher’s exact	0.806
Current immune intolerance protocol					
Yes (%)	5.1%	3.3%	5.3%	Fisher’s exact	0.619
Person who infuses					
Patient (%)	31.1%	31.7%	31.1%	Fisher’s exact	0.817
Caregiver (%)	42.3%	46.7%	41.8%		
Other family caregiver (%)	5.2%	3.3%	5.5%		
Clinical staff (%)	7.0%	3.3%	7.4%		
Visiting nurse (%)	4.7%	6.7%	4.5%		
ER staff (%)	4.4%	3.3%	4.5%		
Other (%)	3.7%	5.0%	3.5%		
Missing (%)	1.6%	0.0%	1.8%		
Venous access device					
Yes (%)	25.0%	33.3%	24.0%	Fisher’s exact	0.155

Bold values represent p<0.05

### Statistical power

Despite the relatively large hemophilia caregiver sample size, the number of caregivers whose care recipients were on extended half-life factor products was relatively small (*n* = 49 out of 448). We implemented a simulation analysis to estimate our statistical power to detect a extended half-life factor product effect given a sample of 49 caring for people only on extended half-life factor product(s) in a total sample of 448. This simulation analysis suggested that we had an estimated 64% power (α = 0.05, β = 0.80) to reject the null hypothesis if it was false [43]. Alternatively, using Cohen’s power primer [42], we had 80% power to detect a large effect size (α = 0.05) with 7 covariates in the model (our models included 12 covariates).

### Clinical correlates of caregiver burden

Correlation analyses examined the relationship between HCI scores and weekly frequency of infusions and bleed frequency over the past 6 months. These analyses revealed that higher social, physical, emotional, financial, and lifestyle impact as well as overall burden had small effect-size correlations [42] with more infusions per week (*r* = 0.15, 0.18, 0.17, 0.16, 0.14, and 0.14, respectively; *p* < 0.02 in all cases). Further, higher physical impact was associated with more bleeds in the past 6 months (*r* = 0.23, *p* < 0.03). T-tests or chi-squared analyses revealed that caregivers in the extended half-life and conventional factor product groups were comparable on age, gender, education, race, marital status, number of children, number of children with hemophilia, number of people providing hemophilia care to, relationship to the care recipient, and number of caregiving years (Tables 1, 2). The conventional group had a higher proportion of hemophilia A patients (*p* = 0.003), a lower proportion of people on a prophylactic regimen (*p* = 0.026), and a higher proportion with a past inhibitor (*p* = 0.046). As could be expected, the hemophilia patients for whom they provided care were different with respect to time on their current factor product and number of infusions per week (*t* = 7.95 and 5.38, respectively; *p* < 0.0001 in both cases). There was a trend such that the patients receiving care were younger in the conventional group (*p* = 0.068). The two groups were comparable on some critical factors: on hemophilia patients’ gender, hemophilia severity, number of bleeds in the last 6 months, number of infusions per month for on-demand treatment, clotting-promoter medications, current inhibitor, on a current immune intolerance protocol, who infuses them, and whether they used a venous access device (Table 2).

### Evaluating extended half-life versus conventional factor products

**Fig. 1:**
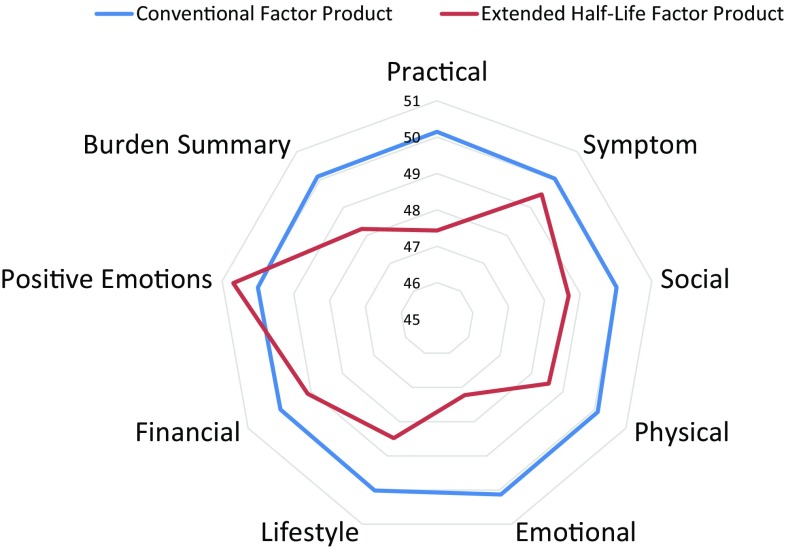
Radar plot showing unadjusted HCI means for extended half-life versus conventional factor product caregivers

Figure 1 shows the radar plot of the mean scores on the HCI subscales and the Burden Summary score for the extended half-life versus conventional factor product caregiver groups. The extended half-life factor group had lower Practical Impact, Emotional Impact, and Burden Summary scores (Effect size = 0.27, 0.29, and 0.19, respectively). The former two are small effect sizes, according to Cohen’s criteria, whereas the latter does not reach Cohen’s criteria for a small effect size [42].

### Regressions evaluating extended half-life versus conventional factor products

Table 3 shows the results of the ordinary least squares multivariable regression models evaluating the impact of extended half-life factor products on the HCI, PedsQL, and WPAI outcomes. These analyses suggest that caring for someone who is on an extended half-life factor product is associated with lower Emotional Impact (β = − 0.11, *p* < 0.05, Adjusted *R*^2^ = 0.06), and shows a trend association with lower Practical Impact (β = − 0.09, *p* < 0.10, Adjusted *R*^2^ = 0.05), after adjusting for relevant covariates. The R-squared statistics associated with these models were consistent with small effect sizes using Cohen’s criteria [42]. There was no association between caring for someone who is on a extended half-life factor product and any other HCI, PedsQL, or WPAI score. Examination of score distributions for the HCI subscales and the Burden Summary revealed that all but one dependent variable was normally distributed: Practical Impact had a skewed score distribution. The PedsQL scores and WPAI did not have skewed distributions. An examination of regression diagnostics revealed that only models predicting Practical Impact and WPAI had heteroscedastic residual distributions (Breusch–Pagan/Cook–Weisberg test for heteroscedasticity chi-squared = 15.30 and 4.20, respectively; *p* < 0.0001 and 0.05, respectively). Log transformations did not render their distributions normal, and did not affect the heteroscedasticity issue. Plots of residuals by covariates suggested that number of caregiving years was a useful weighting variable for the weighted least squares models (i.e., it was continuous not dichotomous, and had a limited range of residuals at low and high values of the variable). Results of the weighted least squares model suggest that caring for someone who is on an extended half-life factor product is associated with lower Practical Impact scores (unstandardized coefficient = − 3.37, *p* < 0.03, Adjusted *R*^2^ = 0.07) (data not shown). Results of the weighted least squares model predicting the WPAI score were consistent with the null findings of the ordinary least squares model (data not shown). The findings remained unchanged when a covariate was added related to whether the caregiver was a parent of the patient to whom s/he provided caregiving support (data not shown).

**Table 3:**
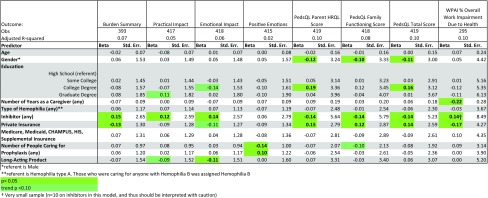
Results of multivariable linear regression models investigating extended half-life versus conventional factor products’ impact on selected HCI, PedsQL, and WPAI scores

### Matched cohort analyses

A cohort-matching analysis (*n* = 377) was done to examine extended half-life versus conventional factor product differences on the three HCI scores that emerged from the mean comparison shown in the radar plot above because they exhibited the largest mean differences (Fig. 1). Sixty-eight caregivers were dropped from the analysis because they could not be matched. This analysis revealed that people on extended half-life factor product tended to have lower Emotional Impact and Practical Impact scores (*t* = − 2.95 and − 2.94, respectively, *p* < 0.05 in both cases). While the differences on the Burden Summary score were lower, it was not statistically significant (Table 4). In terms of Cohen’s effect sizes, the Emotional Impact and Practical Impact were small effect sizes (*d* = 0.30 and 0.28), whereas the Burden Summary score just missed the criterion for a small effect size (*d* = 0.18).

**Table 4:** Matched cohort analyses comparing of those on extended half-life versus conventional factor product groups (*n* = 380) Δǂ

Outcome	Coef.	Std. Err.	*p* value
Burden summary	− 2.20	1.40	0.12
Emotional impact	− 2.95	1.40	**0.04**
Practical impact	− 2.94	1.32	**0.03**

ΔExcluded those on ITI. ǂMatching criteria included if the caregiver was caring for someone on prophylaxis, if they were caring for someone with an inhibitor, the age of the caregiver, and the number of years that they have been a caregiver. Bold values represent p<0.05

## Discussion

Our findings suggest that using extended half-life factor products was associated with a statistically significant effect on reduction of two salient aspects of caregiver burden: the emotional and practical aspects. While maintaining a high level of protection against bleeds, extended half-life factor products appear to be associated with reduced stress, strain, and distress of caregiving. Further, worse social, physical, emotional, financial, and lifestyle impact as well as overall burden were associated with more infusions per week, and worse physical impact was associated with more bleeds in the past 6 months. Additionally, extended half-life factor products reduce the logistic hassles of caregiving, namely the work required to maintain a constant supply of factor product, as well as the effort involved in getting to medical appointments.

These results are promising, and likely the “tip of the iceberg.” Because only 11% of our sample provided caregiving support to people on extended half-life factor products, the study provides an indication of the benefits of such products but the estimate may be low. This work is, however, consistent with a recent systematic review that documented a reduced annualized bleeding rate in hemophilia patients on extended half-life factor products [44]. Further studies should address the long-term benefits to caregivers in terms of reduced negative aspects of caregiving and increased positive aspects of burden. There may also be benefits in terms of societal costs of disease burden [11], such as reduced work and financial impact on caregivers [31] and reduced work disability in patients [45].

This study is the largest study of hemophilia caregivers, and the largest study of hemophilia caregivers with these new extended half-life factor products. It is also the first study to address the impact of hemophilia treatment on caregiver burden, to our knowledge. Nonetheless, our study is limited by its relatively low statistical power to detect a medium effect size (64% power, α = 0.05, β = 0.80) because only 49 out of 448 people in our sample were caring for people on extended half-life factor products. Further, we are unable to characterize the selection bias in this study because of the nature of panel-study and word-of-mouth recruitment: only those opting to participate provide data. Another limitation is that the patients on extended half-life factor products had been using those products for less time as they have only been available since 2014. The effects detected were small to moderate using Cohen’s criteria [42], which makes them likely detectable and important to caregivers [46, 47]. With more experience, the caregivers providing care for patients on extended half-life factor products may show larger effects and / or more effects across the HCI subscales. Statistical power limitations may have affected other detected associations as well. For example, caring for a person with inhibitors was associated with worse overall burden, practical impact, and emotional impact, as well as worse quality of life and work productivity in the multivariable model (Table 3), Further inspection revealed that only 10 of the 295 respondents in that model provided caregiving support to people with inhibitors. This small sample renders the finding less interpretable. Future work should replicate this study and involve larger samples of caregivers providing care to patients on extended half-life factor products, and/or with inhibitors. Alternatively, a longitudinal follow-up of the current sample could address the power problem, both because the impact of such products on caregiver burden may be cumulative over time and because repeated measures can have lower error estimates, thereby increasing the potential to detect important group differences. Future work should evaluate the impact of extended half-life versus conventional factor products on caregiver burden in samples that have had longer experience with the products, as well as the impact over extended follow-up (e.g., 1–5 years of experience with extended half-life factor products). Future work might also focus on parents or caregivers taking care of a child with hemophilia as compared to caregivers of older patients.

In summary, the present study documented a measurable impact of extended half-life factor products on reducing emotional and practical aspects of hemophilia caregiver burden. The detected effects are likely noticeable and important to caregivers, as well as to their care recipient and overall family. The clinical implications of this study are that use of extended half-life factor products may benefit the caregiver and family as well as the patient.
